# Intense visible emission from ZnO/PAAX (X = H or Na) nanocomposite synthesized via a simple and scalable sol-gel method

**DOI:** 10.1038/srep23557

**Published:** 2016-03-24

**Authors:** Y. Zhu, A. Apostoluk, P. Gautier, A. Valette, L. Omar, T. Cornier, J. M. Bluet, K. Masenelli-Varlot, S. Daniele, B. Masenelli

**Affiliations:** 1 Université de Lyon, F-69000 Lyon, France and INL, CNRS, UMR 5270, INSA Lyon F-69621 Villeurbanne, France; 2 Université de Lyon, F-69000 Lyon, France and IRCE Lyon, CNRS, UMR 5256 F-69626 Villeurbanne, France; 3 Université de Lyon, F-69000 Lyon, France and MATEIS, CNRS, UMR 5510, INSA Lyon F-69621 Villeurbanne, France

## Abstract

Intense visible nano-emitters are key objects for many technologies such as single photon source, bio-labels or energy convertors. Chalcogenide nanocrystals have ruled this domain for several decades. However, there is a demand for cheaper and less toxic materials. In this scheme, ZnO nanoparticles have appeared as potential candidates. At the nanoscale, they exhibit crystalline defects which can generate intense visible emission. However, even though photoluminescence quantum yields as high as 60% have been reported, it still remains to get quantum yield of that order of magnitude which remains stable over a long period. In this purpose, we present hybrid ZnO/polyacrylic acid (PAAH) nanocomposites, obtained from the hydrolysis of diethylzinc in presence of PAAH, exhibiting quantum yield systematically larger than 20%. By optimizing the nature and properties of the polymeric acid, the quantum yield is increased up to 70% and remains stable over months. This enhancement is explained by a model based on the hybrid type II heterostructure formed by ZnO/PAAH. The addition of PAAX (X = H or Na) during the hydrolysis of ZnEt_2_ represents a cost effective method to synthesize scalable amounts of highly luminescent ZnO/PAAX nanocomposites.

In ZnO nanoparticles (NPs), visible emission has long been regarded as a drawback to be avoided in order to get intense UV emission. This was mainly driven by the quest of new materials for UV optoelectronics. As it turned out, with the difficulty to p-dope ZnO, that ZnO nanostructures will not soon be used as building blocks for UV optoelectronics, the general opinion on ZnO visible emission has changed. Recent publications have demonstrated that ZnO quantum dots (QDs), either fabricated by the sol-gel method or in a non-thermal plasma reactor, can exhibit visible photoluminescence quantum yield (PL QY) of about 26%^1^ and 60%^2^,^3^, respectively. Using appropriate capping agents such as oleic acid, the PL QY in the blue even reached 76%, but was not stable over more than a few days^4^. The centers responsible for the visible emission are actually so intense that they can be used as single photon emitters^5^,^6^, making these ZnO QDs an interesting alternative to chalcogenide ones, in particular for bioimaging, due to their reduced toxicity and low cost. This intense visible emission can also be useful to build optoelectronic devices such as white LEDs^7^,^8^ or to enhance the conversion efficiency of electrolytic or p-n junction solar cells through the down-shifting process^9^.

Understanding the origins of this visible emission is still not complete, but several reviews have summed up the state-of-the art knowledge^7^,^10^,^11^. The visible emission of ZnO is actually quite broad and consists of several contributions. The implications of impurities (Cu or Li) and intrinsic defects such as O vacancies (V_O_) or interstitials (O_i_), Zn vacancies (V_Zn_) or interstitials (Zn_i_) have been evoked^12^,^13^,^14^. To further illustrate the complexity of the issue, we can mention that the contributions to the visible emission are not simply related to some specific defects, but to defects located at specific locations. For instance, Salviati and co-workers recently showed that V_Zn_ at the (10–10) surface leads to a green emission centered at 2.5 eV (496 nm)^15^. This explains why the visible emission not only depends on the crystalline quality of ZnO nanostructures, but also on their geometry through the presence of specific facets containing specific defects.

For ZnO nanoparticles, the most accepted and used model^16^,^2^ to explain the visible emission is based on the presence of a crystalline point defect close to the surface, acting as an electron trap, and a surface state acting as a hole trap. When a photon is absorbed, giving rise to an electron-hole pair, the electron readily diffuses to the crystalline defect, while the hole gets trapped at the surface. In a second step, the hole tunnels to the electron trap to recombine with the electron and a visible photon is emitted. This model is consistent with experimental observations stating that the PL QY increases as the nanoparticle size is reduced. Indeed, in such a case, the overlap of the electron and hole wavefunctions is increased, leading to an efficient tunneling of the hole to the trapped electron. This model also highlights the crucial role of the particle surface. Therefore, many efforts have been devoted to the control of the surface states. In particular, various polymers have been used to protect the as-synthesized nanoparticles^4^,^17^ or to fabricate particle/polymer nanocomposites with stable, enhanced and tunable photoluminescence^13^,^18^,^10^,^11^.

In this context, post-synthesis surface passivation by polymers has been extensively studied. The addition of polymers during the nanoparticle formation has also been investigated^19^,^20^,^21^. In the latter case the role of the polymer is more complex. When the polymer is an acid or a base, it can act both on the surface charge state (zeta potential, ζ) and on the pH of the solution. The surface charge state is important not only for the control of the hole traps necessary in the aforementioned model, but also to ensure the dispersibility of the resulting particles. On the other hand, the pH value is important to tune the nature of the crystalline defects introduced in the particles and thus the visible emission spectrum. To make it even more complex, the pH value and the ζ potential are linked, ZnO having a zero charge point at pH 9 (the surface charge is positive for pH > 9 values and conversely negative for pH < 9)^1^. Therefore, the addition of polymeric acids (or bases) during ZnO nanoparticle synthesis is a promising and challenging strategy to design QDs having high visible PL QY, that can be competitive with respect to the existing technologies. For further industrial applications, a simple synthesis process providing scalable amount of nanoparticles should be favored.

In this work, we investigate the hydrolysis of ZnEt_2_ (diethylzinc) in presence of a water-soluble weak acid polymer, PAAH (polyacrylic acid), and of mixture thereof with its sodium salt. We demonstrate the effects of the concentration, molecular weight (chain length) and nature of PAAH on the structural and optical (PL QY) properties of the final ZnO nanoparticles. We show that the addition of PAAH during the nanoparticle growth has opposite effects to the ones resulting from its post-synthesis addition. At an optimum concentration, it can actually increase the visible emission efficiency to 20%. Our study also reveals that PAAH with low molecular weight and within a very specific concentration range is to be favored. More interestingly, mixing PAAH with its sodium form PAANa leads to a ZnO/PAA nanocomposite which exhibits an intense visible emission with a PL QY exceeding 50% and even reaching 70% after one month.

## Results

### Effect of the PAAH concentration

**Figure 1:**
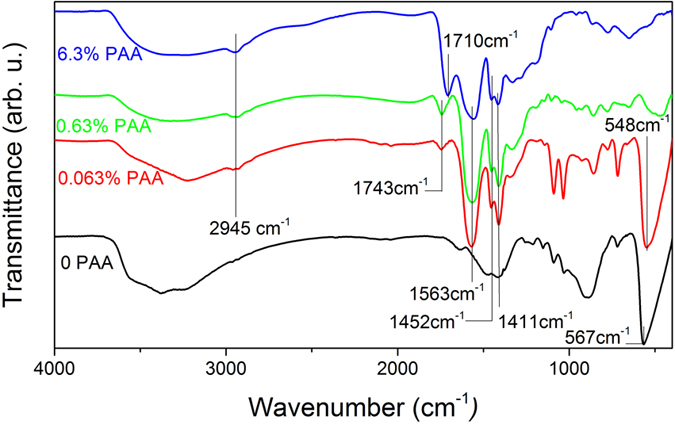
FTIR spectra of ZnO nanoparticles synthesized by the hydrolysis of ZnEt_2_ with different concentrations of PAAH (0 wt%, 0.063 wt%, 0.63 wt% and 6.3 wt% from bottom to top). The plots are shifted along the y-axis for clarity.

PAAH of molecular weight M_w_ = 2000 g mol^−1^ in different ratios (0, 0.063, 0.63 and 6.3 wt% dispersed in water) was added during the synthesis. To ensure that the PAAH interacts with the particles resulting from the hydrolysis of ZnEt_2_, FTIR (Fourier Transform InfraRed) spectroscopy was carried out. The spectra are shown in Fig. 1. In the absence of PAAH, the stretching mode of the Zn-O bond^14^ can be seen in the range spanning from 540 to 570 cm^−1^. The broad feature around 3400 cm^−1^ corresponds to the stretching mode of the O-H bond, indicating the presence of hydroxide groups in all the samples. When the PAAH concentration increases, the Zn-O stretching mode is progressively reduced, being hardly distinguishable with 0.63 wt% of PAAH and absent at 6.3 wt % of PAAH. This shows that a high concentration of PAAH during the synthesis tends to degrade the crystalline quality of the resulting ZnO particles. Additional peaks at 2945 cm^−1^, 1563 cm^−1^, 1452 cm^−1^, and 1411 cm^−1^ are related to PAAH. In particular, the peaks at 1563 cm^−1^ and 1411 cm^−1^ correspond to the asymmetric (*v*_as_) and symmetric (*v*_s_) modes of the carboxylate anion (COO^−^), respectively. The Δ(*v*_as_−*v*_s_) value of 152 cm^−1^ indicates that the interaction between PAAH and ZnO occurs probably through bridging/chelating modes, as evoked in ref. 22. However, with 0.063 wt% and 0.63 wt% of PAAH, a band at 1743 cm^−1^ is observed, which shifts to 1710 cm^−1^ with 6.3 wt% of PAAH. These bands at 1743 and 1710 cm^−1^ correspond to the C = O stretching mode for free carboxylic acid (COOH) group and hydrogen-bonded COOH group, respectively^23^. We can first conclude that a minor part of PAAH is still in its protonated form for low PAAH concentrations, but increases when the PAAH concentration reaches 6.3 wt%. Furthermore, we can infer that at high concentrations, the excess of PAAH links to the rest of PAAH through H bonds.

**Figure 2:**
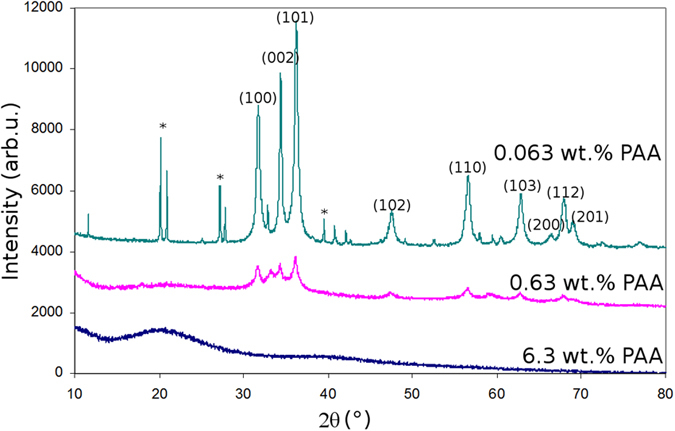
XRD diffractograms of ZnO nanoparticles synthesized by the hydrolysis of ZnEt_2_ with different concentrations of PAAH (0.063 wt%, 0.63 wt%, and 6.3 wt% from top to bottom). The plots are shifted along the y-axis for clarity. Peaks labeled *refer the Zn(OH)_2_ wulfingite phase.

To get more insight into the crystalline quality of the samples, we performed X-Ray diffraction analysis (XRD). The diffractograms are presented in Fig. 2. A clear trend is observed: as the PAAH concentration increases, the intensity of the diffraction peaks associated with the ZnO wurtzite structure decreases and the peaks eventually disappear at the highest concentration. This is in accordance with the FTIR results and confirms that the crystalline quality is degraded as the PAAH content increases. Consequently, more defects are gradually introduced in ZnO particles to eventually lead to an amorphous material. Concomitantly, the size of the particles estimated using the Debye-Scherrer equation, is reduced from 17 nm to 8 nm. It is worth noting that at the concentration of 0.063 wt%, some traces of ε-Zn(OH)_2_ (wulfingite) are present along with the wurtzite ZnO phase^24^,^25^.

**Figure 3:**
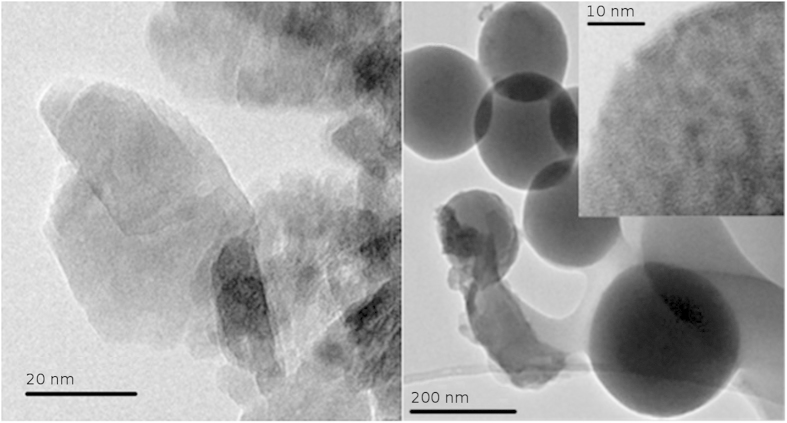
TEM images of ZnO nanoparticles synthesized by the hydrolysis of ZnEt_2_ with 0.063 wt% of PAAH (left) and with 0.63 wt% of PAAH (right); the inset is a zoom of the surface of a sphere.

The PAAH addition during the growth not only controls the size of the nanoparticles but also their shape, as illustrated in the TEM images of Fig. 3. When no or a little amount (0.063 wt%) of PAAH is added, crystalline platelets of about 20 nm are formed (cf. Fig. 3 a) and Fig. S1 from the supporting information). The dimension of these platelets is difficult to measure accurately because of their superposition but their apparent size seems in accordance with the crystalline domain size estimated from the XRD diffractograms of Fig. 2. When 0.63 wt% of PAAH is added, spheres of about 200 nm in diameter are observed. As shown in the inset of Fig. 3b), these spheres are composed of nanoparticles embedded in a polymeric matrix. The TGA (thermogravimetric analysis) data up to 700 °C demonstrates that the inorganic/organic weight ratio is around 60/40 (cf. Fig. S2 and Table S1 from the supporting information). From the TEM images, the nanoparticles are crystallized (cf. Fig. S3 from the supporting information) and made of ZnO. They are smaller than 10 nm, in accordance with the ZnO crystalline domain size estimated from the XRD diffractograms. It thus seems that when the PAAH content increases during the synthesis, the resulting ZnO nanoparticles turn from nanoplatelets to hybrid supramolecular mesospheres^21^. At the highest PAAH concentration of 6.3 wt%, it is difficult to assess the microstructure of the spheres. Nevertheless, it is unlikely that they consist of crystalline ZnO nanoparticles embedded in PAAH matrix.

**Figure 4:**
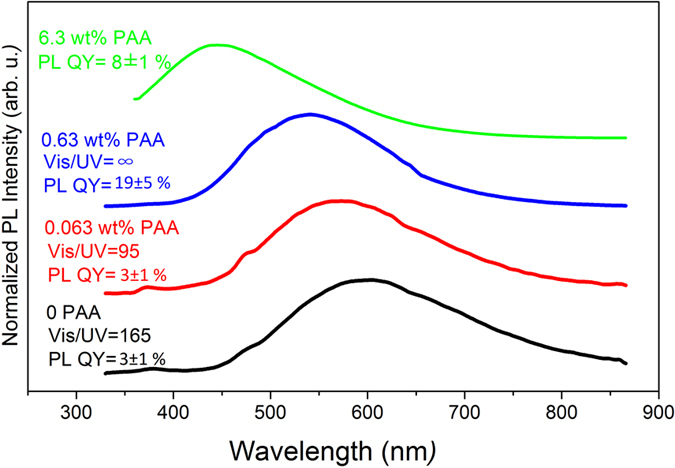
PL spectra of ZnO nanoparticles synthesized by the hydrolysis of ZnEt_2_ with different concentrations of PAAH (bottom curve: 0 wt% of PAAH; lower middle curve: 0.063 wt% of PAAH; higher middle curve: 0.63 wt% PAAH and top curve: 6.3 wt% of PAAH). The plots are shifted along the y-axis for clarity.

Regarding the corresponding optical properties, the PL spectra are shown in Fig. 4. When the PAAH concentration increases from 0 to 0.063 wt%, both the visible/UV intensity ratio (marked as Vis/UV in Fig. 4) and the PL QY remain low. The addition of PAAH at such a low content has almost no influence on any property of the resulting material. As it increases to 0.63 wt%, the Vis/UV ratio tends to infinity (i.e. there is no UV emission) and the PL QY rises dramatically from 3 to 19%. The PL QY then decreases to 8% when the PAAH concentration increases to 6.3 wt%. The PL QY evolution is accompanied with a gradual blue-shift of the PL emission, from 600 nm to 540 nm at 0.63 wt% PAAH and to 450 nm at 6.3 wt%. This blue shift is not due to the appearance of the PL from the PAAH itself. As can be seen in Fig. S4, the PL emission of PAAH is characterized by a sharp feature at ~460 nm which is not present in the spectra of the samples. The observed blue shift may rather indicate a change in the nature of the defects present in ZnO NPs, with a decrease of the defects responsible for the visible emission above 450 nm.

**Figure 5:**
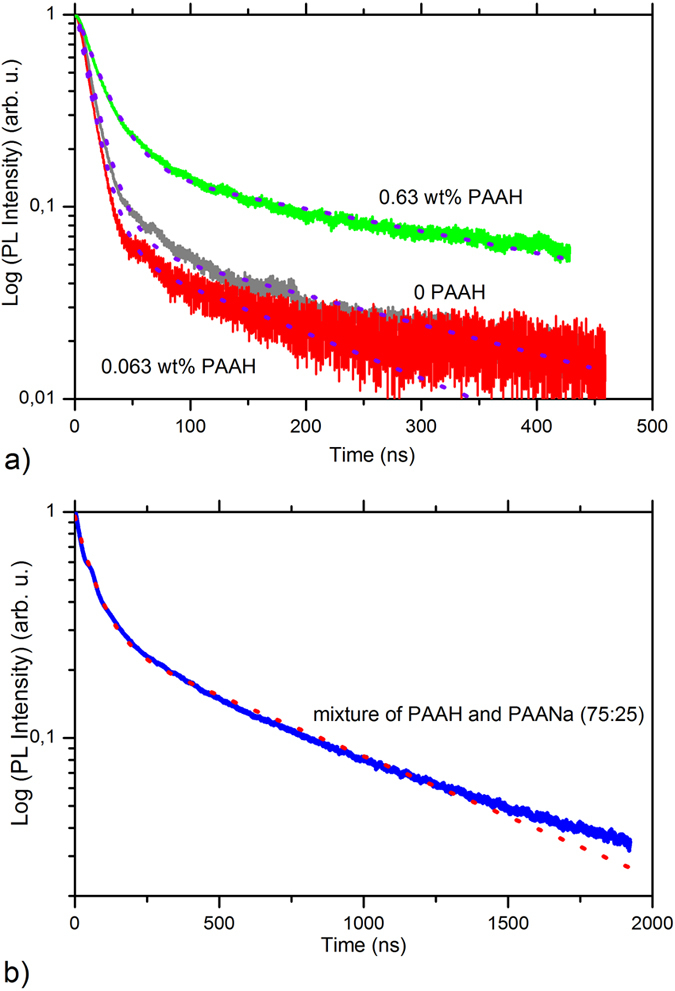
TRPL decay curves of ZnO nanoparticles synthesized by the hydrolysis of ZnEt_2_ with no PAAH, with 0.063 wt%, 0.63 wt% of PAAH (**a**) and the mixture of PAAH with PAANa (0.63 wt%, 75:25) (**b**). The peak at 6 ns is an artefact from the acquisition set-up. Fitting results to the bi-exponential decay are shown in Table 1.

The dynamics of the PL emission has been studied by TRPL (Time Resolved Photoluminescence) measurements. The results are presented in Fig. 5 and summed up in Table 1. The emission is characterized by two components, a rapid one and a slow one, indicating that two distinct populations are involved in the visible luminescence process. With no PAAH or with a small amount of PAAH, the rapid component has a decay time τ_1_ of about 13 ns, while the slow one is one order of magnitude slower (τ_2_ ~ 200 ns). When 0.63 wt% of PAAH is used, both decay times increase to 20 ns and 341 ns. This is in accordance with the increase in the PL QY and tends to show that the non-radiative centers are in lower concentration.

**Figure 6:**
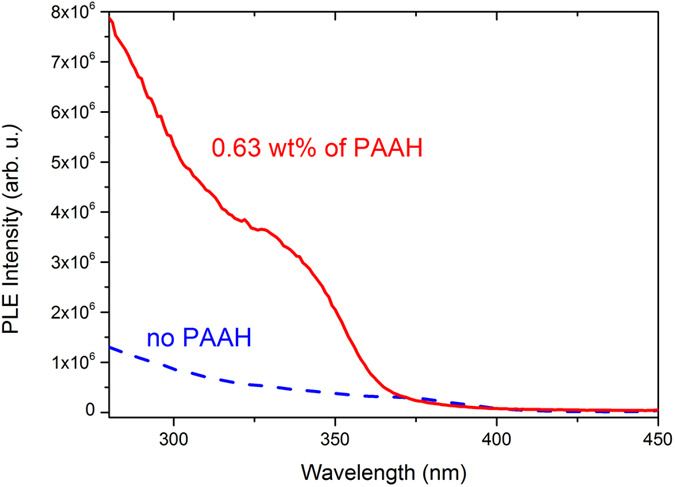
PLE spectra of ZnO nanoparticles synthesized by the hydrolysis of ZnEt_2_ with no PAAH and with 0.63 wt% of PAAH.

PL excitation (PLE) spectroscopy was performed to investigate the de-excitation channels among the samples. The results for samples synthesized with 0 and 0.63 wt% of PAAH are shown in Fig. 6. For ZnO nanoparticles synthesized without PAAH, the excitation threshold corresponds to the gap of bulk ZnO. For the particles synthesized with 0.63 wt% of PAAH, the global intensity of the signal is higher (in accordance with the higher PL QY). Moreover, a new resonant feature is observed, peaking at around 340 nm (3.65 eV) and starting approximately at 365 nm (3.4 eV). This feature is in accordance with the optical bandgap measurement as obtained from the UV-VIS reflection spectrum via Kubelka-Monk approximation (Fig. S5) which shows a clear threshold at this value. It is most probably not related to ZnO, since the nanoparticles are still too large to undergo a significant blue shift due to the quantum confinement effect. On the contrary, it can originate either from Zn(OH)_2_^26^ or from the PAAH. At this point, it has to be mentioned that no crystalline zinc hydroxide compound has been detected in this sample. Moreover, as shown in Fig. S6, the PLE spectrum of PAAH actually presents a broad resonance at 343 nm. We thus conclude that this resonance in the PLE spectrum is probably related to the presence of PAAH.

**Figure 7:**
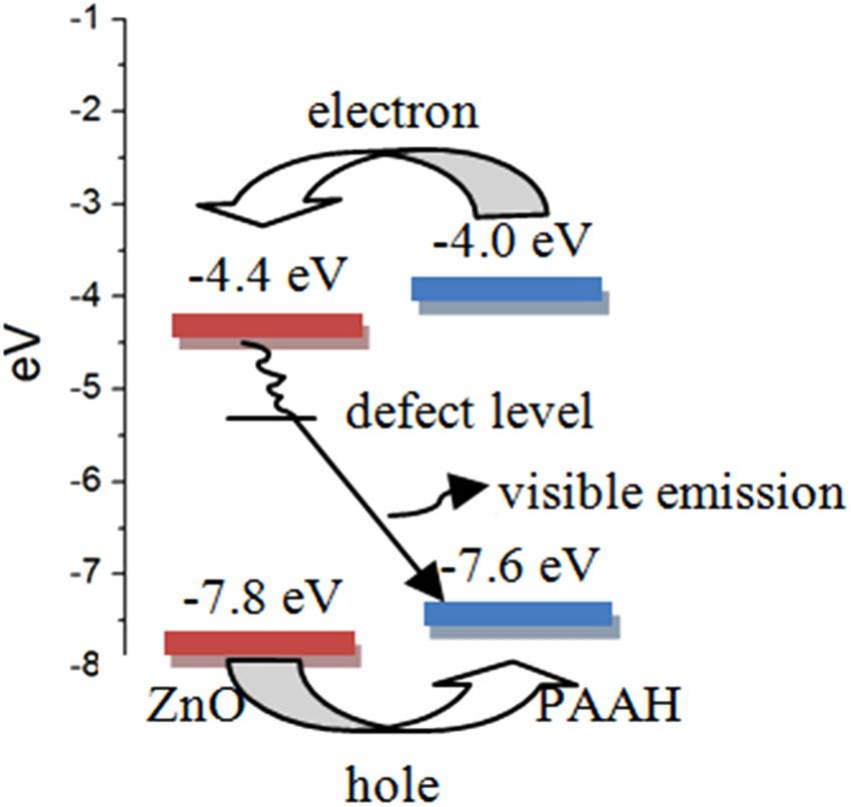
Schematic diagram of the energy levels for ZnO/PAA nanocomposites and supposed mechanism of PL QY enhancement.

On the basis of these observations, we propose a model for the energy diagram of the ZnO/PAA composite. Figure 7 shows the energy diagram of the composite, with the conduction and valence band levels of ZnO as well as the HOMO (highest occupied molecular orbital) and LUMO (lowest unoccupied molecular orbital) of PAAH. Both materials form a type II heterojunction. When a high energy photon is absorbed either in the ZnO part or in the PAAH part, the electron readily thermalizes at the bottom of the ZnO conduction band while the hole gets trapped at the HOMO of PAAH. This mechanism limits the formation of the exciton in ZnO if both materials are intermixed on a length scale smaller than the exciton diffusion length. Afterwards, the electron gets trapped at the defect level in the ZnO gap, responsible for the visible emission (presumably related to V_Zn_ or O_i_ which are the most likely point defects in our O-rich synthesis method^26^). Eventually, the trapped electron and hole recombine to give rise to the visible emission. The emission efficiency is increased because of the strong overlap between the wavefunctions of the trapped electron and hole.

To sum up the effect of the PAAH concentration during the synthesis, there is clearly an optimum concentration around 0.63 wt% which leads to small (~8 nm) ZnO nanocrystals embedded in large (~200 nm) polymeric spheres. The PL QY reaches almost 20% at the maximum. For lower PAAH contents, almost no difference is observed with respect to the samples synthesized without PAAH. When the PAAH concentration is high (6.3 wt%), no indication of crystalline ZnO is obtained and the PL emission is strongly blue-shifted and presents a reduced QY.

The reason for the PL QY enhancement is not completely clear. It should be stressed that the effect of PAAH during synthesis is inverse to its effect when added after the synthesis. Indeed, when added after the growth, PAAH acts a passivating capping agent^27^,^28^, leading to a reduced visible emission and an enhanced UV one. Several reasons can be evoked to explain our observations. First, we have established that the addition of PAAH reduces the size of the particles. In the generally accepted model, as mentioned previously, the size reduction is beneficial to the PL QY. However this is significant for very small sizes (~2 nm in diameter). In our case, the nanoparticles remain “large” with respect to the required dimension. Thus this explanation most probably does not hold in our case. Second, according to the observed crystalline quality degradation, we can assume an increase in the concentration of the crystalline defects responsible for the visible emission, or as written above, an enhanced efficiency of the coupling between these defects and the HOMO of PAAH. It has been demonstrated that PAAH interacts more strongly with the non-polar facets of ZnO^29^. We can thus assume that PAAH favors their presence. Since these surfaces are beneficial to the visible emission, the increase in the visible PL QY can be explained at least partially. Through the chelation by carboxylic groups of Zn on the nanoparticles, some V_Zn_ complexes, beneficial to the visible luminescence, may be produced. Eventually, the fact that ZnO particles are embedded in polymer spheres of a few hundreds of nm in diameter may also be beneficial. Such spherical structures, resulting from the aggregation of nanoparticles, has been reported several times^30^,^31^ in sol-gel fabrication of ZnO nanoparticle-based transparent electrodes for dye sensitized solar cells. In these studies, the hierarchical spherical aggregates are reported to increase light scattering and thus its trapping and absorption in the film. Apart from the size reduction, the aforementioned mechanisms are likely to cooperate to lead to a high PL QY in the film synthesized with 0.63 wt% of PAAH.

### Effect of the polymer molecular weight

**Figure 8:**
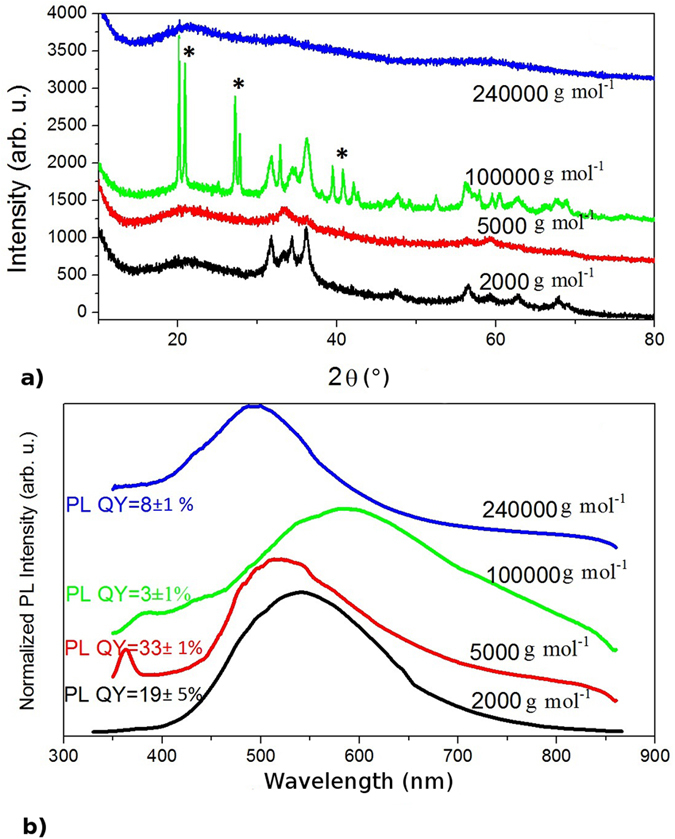
XRD diffractograms (**a**) and PL spectra (**b**) of ZnO nanoparticles synthesized by the hydrolysis of ZnEt_2_ with different lengths of PAAH of 0.63 wt% (240,000, 100,000, 5,000 and 2,000 g mol^−1^ from top to bottom). The plots are shifted along the y-axis for clarity. Peaks labeled *refer the Zn(OH)_2_ wulfingite phase.

**Figure 9:**
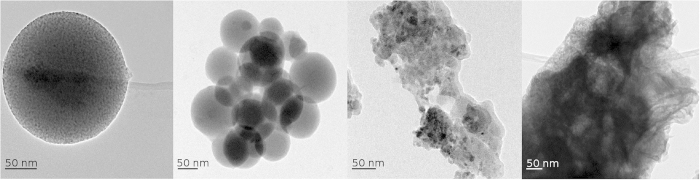
From left to right, TEM images of ZnO nanoparticles synthesized by the hydrolysis of ZnEt_2_ with PAAH of 0.63 wt% and molecular weight of 2,000 g mol^−1^, 5,000 g mol^−1^, 100,000 g mol^−1^ and 240,000 g mol^−1^.

PAAH with different molecular weights (2,000; 5,000; 100,000 and 240,000 g mol^−1^) has been added at the optimal concentration of 0.63 wt% during the synthesis to get ZnO/PAAH nanocomposites. The molecular weight of PAAH influences the nanocomposite morphology and properties, as shown in the XRD diffractograms of Fig. 8, and this in an unusual manner. While a molecular weight of 2,000 g mol^−1^ leads, as already mentioned, to small (~8 nm) wurtzite nanoparticles in polymer spheres, the use of chains of 5,000 g mol^−1^ leads to a diffractogram often seen in disordered ZnO or Zn(OH)_2_^32^. The two peaks at 33,4° and 60° are also found in the diffractograms of small hydrozincite particles (Zn_5_(OH)_6_(CO_3_)_2_)^33^,^34^ but the latter phase is unlikely to occur in our synthesis process. When the molecular weight of the PAAH is increased to 100,000 g mol^−1^, the wurtzite structure is recovered and the corresponding size is about 8 nm. However, additional peaks corresponding to the ε-Zn(OH)_2_ compound (wulfingite) are clearly seen. For a molecular weight of 240,000 g mol^−1^, the sample is amorphous. Here again it is instructive to compare the XRD data to the TEM ones, presented in Fig. 9. As discussed previously, for a molecular weight of 2,000 g mol^−1^, the sample consists in large polymer spheres (around 200 nm) embedding small ZnO nanoparticles. At a molecular weight of 5000 g mol^−1^, the morphology is similar, but mesospheres are smaller in size (around 100 nm) (Fig. 9b). For a molecular weight of 100,000 g mol^−1^, nanocrystalline ZnO particles of 8–10 nm in size are observed in a polymer matrix without any specific shape (Fig. 9c). At the highest molecular weight of 240,000 g mol^−1^, no ZnO particle is observed and it seems that the polymer matrix makes flakes instead of spheres (Fig. 9d). The BET and TGA data show no general trend and, in agreement with the XRD data, the sample obtained for the molecular weight of 100,000 g mol^−1^ displays peculiar properties since it gives the highest specific surface area (51 m^2^ g^−1^) and the lowest weight loss between 150–500 °C (32%) so the lowest organic content compared to others (Fig. S2 and Table S1).

The corresponding optical properties are obviously affected by the PAAH molecular weight as can be seen on Fig. 8b). As the PAAH weight increases from 2,000 to 5,000 g mol^−1^, the nanocomposite is made of mesospheres, but has an overall inferior crystalline quality and the PL QY rises from 19 to 33%. A UV emission appears at 363 nm while the visible emission shifts from 540 nm to 516 nm. The excitonic emission peak is blue-shifted with respect to the bulk one (~380 nm). This shift may be attributed to the quantum confinement effect. Assuming that this excitonic emission comes from quantum confined ZnO amorphous nanoparticles, we estimate the size of the quantum dots to be about 5 nm^35^,^36^. Moreover, the blue shift of the visible emission is consistent with this observation, since it has been shown by Yin *et al.*^37^ that the deep levels in the gap related to the visible emission are also subject to the quantum confinement effect. When the PAAH weight increases to 100,000 g mol^−1^, the PL spectrum and PL QY (3%) are identical to those of ZnO nanoparticles synthesized without PAAH. In this case, the optical properties of ZnO/PAAH nanocomposites are not influenced by the presence of PAAH because the reaction between PAAH with longer carbon chains (corresponding to higher weights) and ZnO nanoparticles becomes weaker^38^. A much higher PAAH weight (240,000 g mol^−1^) induces amorphous nanocomposites with a PL spectrum shifted to the blue range and a low PL QY (8%), as in the case of a high concentration of PAAH (Fig. 4, PAAH weight of 2,000 g mol^−1^). This is consistent since at high concentration of small chained PAAH or at low concentration of long chained PAAH, the steric hindrance caused by PAAH is of the same order of magnitude and limits the hydrolysis and nucleation of ZnO nanoparticles. Consequently, as far the PL QY value is concerned, a low molecular weight of PAAH should be favored.

### Effect of the polymer nature

To examine the ZnO/PAAH nanocomposites formation mechanism, PAAH has been substituted with 0.63 wt% of PAANa (M_w_ = 4000 g mol^−1^). The FTIR spectrum of the ZnO NPs synthesized with PAANa (Fig. S7a) shows the presence of deprotonated carboxylate group (COO^−^) modes (at 1568 and 1404 cm^−1^), but their amount is much smaller than that of OH groups, when compared to the PAAH addition case. Also, the ZnO stretching mode is clearly visible indicating a good crystallinity. This is confirmed by the Raman spectrum of the sample which shows the usual modes of the wurtzite structure. In fact, the Raman spectrum, PL spectrum and PL QY of the ZnO/PAA nanocomposite synthesized using PAANa are similar to those synthesized without surfactant (Fig. S7b–d). They both have the crystalline wurtzite structure. Consequently, the presence of H is beneficial to the synthesis of highly luminescent disordered nanocomposites. We can also notice that the difference in wavenumber of the COO^−^ modes is larger in the present case than in the case of PAAH addition. This reveals that the interaction of the carboxylate group is closer to a mono-dentate bond in the present case, whereas it adopts a bi-dentate (or bridging) interaction when PAAH is used^23^. The nature of PAA therefore controls the surface of ZnO particles.

**Figure 10:**
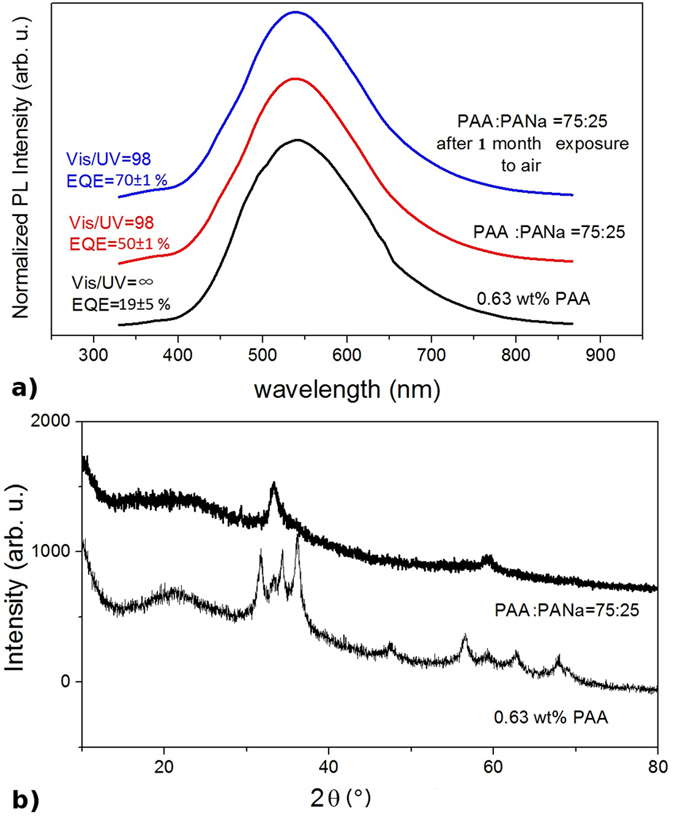
(**a**) PL spectra of ZnO nanoparticles synthesized by the hydrolysis of ZnEt_2_ with 0.63 wt% of PAAH only (bottom curve) and with PAAH and PAANa of 3:1 (middle curve) and after 2 months exposure to air (top curve). (**b**) XRD diffractograms of ZnO nanoparticles synthesized by the hydrolysis of ZnEt_2_ with 0.63 wt% of PAAH only (bottom curve) and with PAAH and PAANa of 3:1 (top curve). The plots are shifted along the y-axis for clarity. (**c**) HRTEM of small ZnO nanocrystals. The wurtzite structure is clearly seen from the inset showing the FFT of the image.

Mixing PAANa with PAAH (ratio of 1:3) of optimal 0.63 wt% is found to be highly beneficial for the visible emission. While the PL spectrum does not change, the PL QY increases to 50% and even higher to 70% after a one month exposure to air (Fig. 10a). This QY increase over one month can be explained by OH adsorption and diffusion among the sample. Such an effect has already been reported^2^ and OH groups are known to increase the visible emission of ZnO. However, no synergetic effect such as the one of PAAH and PAANa has been reported to the best of our knowledge. The XRD diffractogram of the resulting sample does not reveal any wurtzite phase. On the contrary, as for the sample synthesized with pure PAAH of M_w_ = 5,000 g mol^−1^, it points out the presence of highly disordered ZnO or Zn(OH)_2_ or, less likely, hydrozincite particles in an amorphous material (cf. Fig. 10b). Besides, the HRTEM of this sample, shown in Fig. 10c and S8 of the supporting information, reveals the unambiguous presence of small ZnO nanocrystals. Among the observed nanocrystals, there might be some Zn(OH)_2_ ones as well. It is however difficult to be categorical since this assumption is based on the detection of crystalline planes separated by a distance of 2.7 Å. They may correspond to the (112) plane of wulfingite Zn(OH)_2_ but they could be attributed to slightly distorted (0002) planes of wurtzite ZnO as well, given the accuracy of the FFT. Nevertheless, we can confirm the presence of very small (~2 to 3 nm) ZnO nanocrystals surrounded by polymer ligands. The small size of the particles is in accordance with the very high PL QY measured since it has been established that high QY are obtained at such small sizes^2^.

The exact interaction is not straightforward to describe. However, adding PAANa modifies the dissociation of PAAH written in equation (1) and shifts the equilibrium to the left hand side term (buffer medium). As stated above, this will impact the shape of the nanoparticles, the nature of the planes constituting the nanoparticles, as well as their crystalline quality.


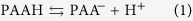


It thus seems that the interaction of the protonated form PAAH with the precursor (ZnEt_2_) is beneficial as far as luminescence is concerned.

TRPL has been performed on this sample and the time trace is shown in Fig. 5b and the result of the fitting procedure is reported in Table 1. For this sample the fast component is now characterized by a decay time τ_1_ of 65 ns, while the slow component has a decay time τ_2_ of 787 ns. Both decay times are increased, indicating a reduced effect of the non-radiative centers. In the literature, three components with decay times differing by at least one order of magnitude have been reported. Typically, a fast component (<10 ns) is observed for the emission in the blue. The green visible emission is characterized by an extremely long decay time between 1340 ns^16^ and 1850 ns^18^. Eventually, as mentioned above, a third intermediate component is reported with a decay time of 60 ns, but it has not been assigned definitely. In our case, we observe only two components (no blue emission), the shorter one with a decay time of a few tens of ns and a longer one of several hundreds of ns. The time τ_2_ measured here could be related to the long decay time already reported, but reduced to a lower value due to the presence of quenching centers. Concerning the fast component, its decay time is of the same order of magnitude as that of the unassigned contribution reported in the literature at 60 ns^18^. If the long decay time contribution can be explained by the commonly accepted model, the second one cannot. The latter is likely related to hybrid states at the interface between ZnO and the organic acid. Nevertheless, since the PL QY of these samples is quite high, the relaxation of electrons and holes is mainly ruled by the radiative recombination. Therefore, the reported decay times are close to the nominal values of the radiative processes involved in the visible emission in our ZnO nanoparticles.

## Conclusions

In conclusion, we demonstrate that the hydrolysis of ZnEt_2_ in the presence of PAAH or a 3:1 mixture of PAAH + PAANa is a cost effective strategy to synthesize scalable amounts of highly luminescent surface-modified ZnO/PAAX (X = H, Na) nanocomposites. The effects on the ZnO luminescence of the PAAH addition during the synthesis are opposite to those of ZnO capped by PAAH. In particular, the UV emission is suppressed while the visible emission is increased. The key parameter lies in the control of the degree of crystalline disorder in the ZnO particles embedded. A rather large degree of disorder is required for a high PL QY. This control can be obtained first by tuning the PAAH concentration. Below an optimal concentration, the addition of PAAH has no significant effect whereas above this optical concentration, too much disorder is induced, leading to a completely amorphous material. The molecular weight of PAAH molecules has also a major impact. Low molecular weights (2,000–5,000 g mol^−1^) appear more efficient to achieve a high PL QY. More interestingly, a mixture of PAAH and PAANa (3:1) is extremely favorable for an intense visible emission. The PL QY rises up to 50% and even to 70% over one month, making this material an interesting alternative to existing high QY luminescent nanomaterials. Several mechanisms may be involved in this enhancement. We have observed in particular that PAAH leads to the formation of hybrid nanocomposite mesospheres. This geometry may be beneficial for light-matter interaction. Moreover, a mixture of short length PAAH/PAANa can lead to small (~2 nm) ZnO nanocrystals for which the PL QY is expected to be large.

To get more insight into this enhancement, a model based on the hybrid type II heterostructure formed by ZnO/PAAH is proposed and the corresponding decay times for radiative recombination are estimated. Because of the commercial availability and low cost of the reactants, we are currently investigating the up-scaling (10–100 L batch) of those hybrid systems in order to highlight their promising potential for industrial applications (photovoltaics, LED, cosmetics).

## Methods

ZnEt_2_ solution at 15 wt% in toluene, polyacrylic acid (PAAH, 2,000 g mol^−1^) aqueous solutions at 63 wt% and pure PAAH (5,000; 100,000 and 240,000 g mol^−1^) were purchased from Sigma-Aldrich. Sodium polyacrylic acid salt (PAANa, 4000 g mol^−1^) aqueous solution at 52 wt% was purchased from Coatex company (Genay, France). All reactants were used as received.

In a typical procedure, 25 ml of water with or without PAAH and/or PAANa was placed in a Schlenk tube. In order to modify the surface state of resulting ZnO nanoparticles, PAAH of 2000 g mol^−1^ was added to water at different concentrations, namely 0.063, 0.63 and 6.3 wt%. These solutions were obtained by diluting 1000, 100 and 10 times respectively the initial solution at 63 wt%. Different molecular weights of PAAH (5000, 100000 and 240000 g mol^−1^) and a mixture of PAAH + PAANa (3:1) at 0.63 wt% were also studied. In these cases, the starting products were pure PAAH or PAANa. Their amount was weighted so that their weight in the water solution amounted to 0.63 wt% in total. Especially, when both PAAH and PAANa were added, the sum of their respective weights amounted to 0.63 wt% of the water solution. Subsequently, 1.8 ml (2.6 mmol) of the ZnEt_2_ solution were added dropwise under a vigorous stirring. The resulting ethane was removed via a bubbler. ZnEt_2_ being pyrophoric, the reaction was carried out in an inert atmosphere^27^. For all experiments, stirring was continued overnight at room temperature. The ZnO formed was subsequently isolated by centrifugation at 4000 rpm for 10 minutes and then the isolated white powder was washed with ethanol (20 ml) and rinsed twice in water. Finally, the compound was placed at 70 °C for 4 hours.

A Bruker (Siemens) D5005 diffractometer using the K-alpha radiation of Cu (1.54184 Å) permitted to probe the crystalline structure via X-ray diffraction (XRD) analysis. The NP size and crystallographic structure were analyzed using Transmission Electron Microscopy (TEM JEOL 2010F). The chemical composition was investigated by FTIR (Fourier Transform Infra-Red) spectroscopy (Vertex 80 Bruker Optics). TG-DTA (thermogravimetric-differential thermal analysis) data were collected in air using a Setaram TGA92-12 thermal analyzer (thermal ramp 10 °C min^−1^, temperature range 20–600 °C). Nitrogen adsorption–desorption isotherms were collected at 77 K using a Micromeritics ASAP 2020 apparatus. Prior to the measurement, samples were desorbed at 100 °C for 6 h under vacuum. The Brunauer–Emmett–Teller (BET) method was used to calculate the specific surface areas from the adsorption data in a relative pressure range from 0.05 to 0.25.

Photoluminescence (PL) spectroscopy was performed with a set-up consisting of a continuous laser excitation at 266 nm (1.2 mW, Crylas FQCW 266-10). The emission was dispersed by a spectrometer (iHR Triax 320 Jobin-Yvon) and detected by a liquid-nitrogen cooled CCD detector. PL Quantum yield (PL QY) was measured using an integrating sphere and the same excitation and detection set-up as for the PL measurements. The PL QY was calculated according to the procedure developed by Friend *et al.*^39^ Calibration of the integration sphere was performed using two samples of known high and low PL QY: a colloidal solution of CdSe quantum rods with a PL QY of 80% and a porous silicon sample of 3.2% PL QY. For PL and PL QY measurements, ZnO nanoparticle powders were deposited onto an indium foil as a substrate. Photoluminescence excitation (PLE) spectra were measured using a FLS920 Series fluorescence spectrometer from Edinburgh Instruments (Livingston, UK). A 450-W Xe900 continuous xenon arc lamp was used as the excitation source. For the Time Resolved photoluminescence (TRPL) measurements, a 266 nm laser (average power of 10 mW with 10 ns pulse duration and 20 Hz repetition rate) was used as the excitation source. The emission was dispersed using a Jobin–Yvon HR 640 monochromator and detected with a GaAs (Hamamatsu H8567–03) photomultiplier tube and registered using an oscilloscope sampling at 2 gigacounts per second.

## Additional Information

**How to cite this article**: Zhu, Y. *et al.* Intense visible emission from ZnO/PAAX (X = H or Na) nanocomposite synthesized via a simple and scalable sol-gel method. *Sci. Rep.* **6**, 23557; doi: 10.1038/srep23557 (2016).

## Supplementary Material

Supplementary Information

## Figures and Tables

**Table 1:** Time constants obtained by fitting TRPL spectra in Fig. 10 with the biexponential decay and the relative contributions of each exponential function.

Surfactants addition	τ_1_ [ns]	Relative amplitude	τ_2_ [ns]	Relative amplitude
No surfactants	13	0.93	243	0.07
0.063 wt% PAAH	12	0.96	184	0.04
0.63 wt% PAAH	20	0.84	341	0.16
Mixture of PAAH and PAANa (3:1)	65	0.70	787	0.3
