# Supplementary material for: Intense visible emission from ZnO/PAAX (X = H or Na) nanocomposite synthesized via a simple and scalable sol-gel method
Source: Sci Rep. 2016 Mar 24;6:23557. doi: 10.1038/srep23557 (PMC4806374; doi:10.1038/srep23557)
Supplement: Supplementary Information [file srep23557-s1.pdf]

Supporting Information

Intense visible emission from ZnO/PAAX (X = H or Na) nanocomposite synthesized via a simple and scalable sol-gel method

Yao Zhu, Aleksandra Apostoluk, Pierrick Gautier, Audrey Valette, Lama Omar, Thibaut Cornier, Jean-Marie Bluet, Karine Masenelli-Varlot, Stephane Danièle and Bruno Masenelli**

Further general methods.

Beside the XRD analysis, the crystalline structure was probed by Raman spectroscopy (Raman Thermo-Fisher DXR) with the laser excitation at 532 nm and intensity of 10 mW. Furthermore, the UV-Visible diffuse reflectance allows the determination of the bandgap. In order to get the optical bandgap our samples, we applied this method using the Avalight-DH-S apparatus providing a continuous spectrum from a deuterium and halogen source within a range of 200 nm to 2500 nm. The reflected light was collected via a bundle of six optical fibers, while the seventh fiber allowed the collection of the radiation non-absorbed by the material. The acquisitions were made from 300 to 800 nm, allowing the determination of the band gap energy of the studied samples via the Kubelka-Monk approximation.

Figure S1: TEM images of the ZnO/PAAH composite synthesized by the hydrolysis of $ZnEt_2$ using a solution of 0.063 % of PAAH.

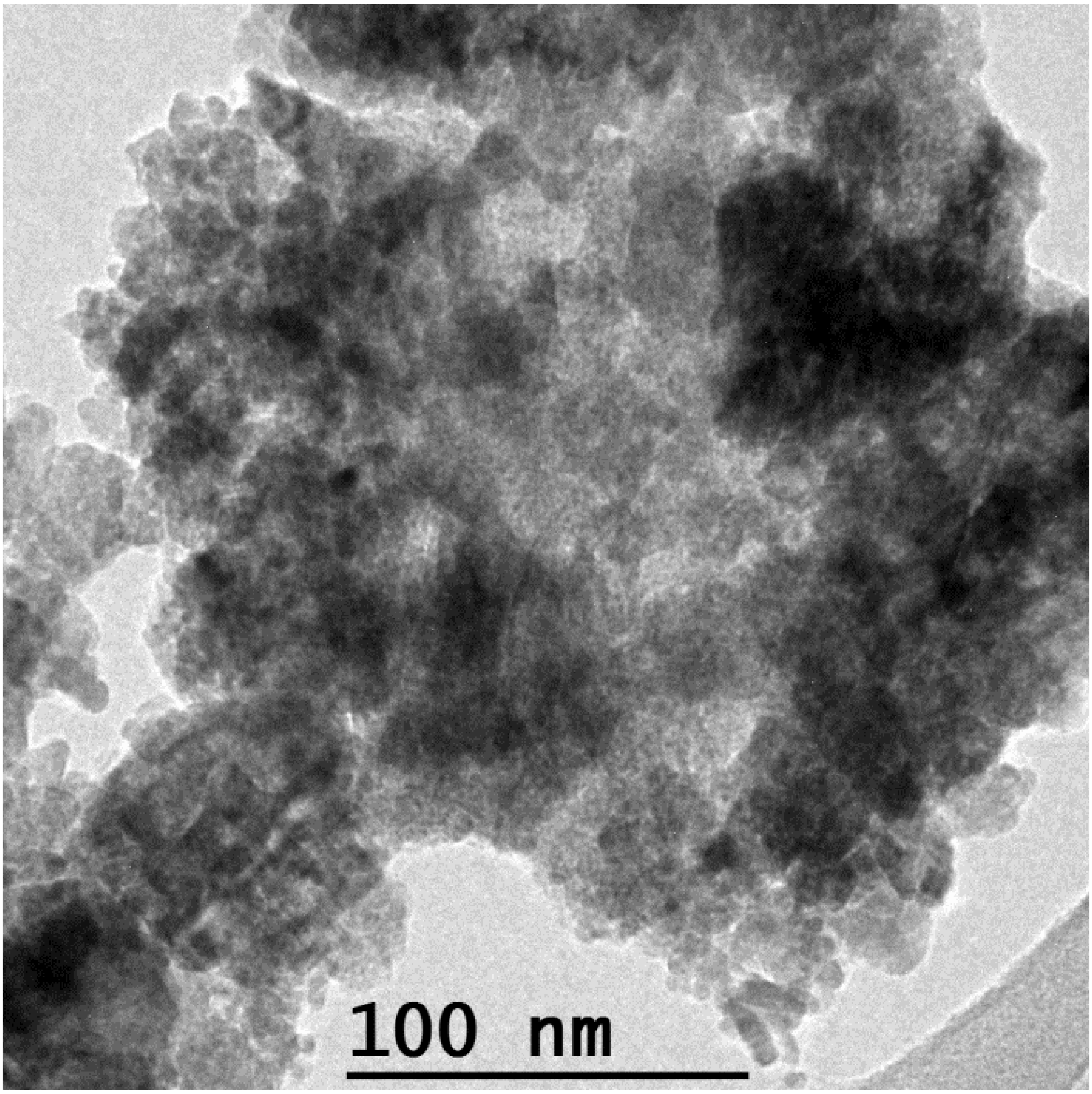

Low magnification image showing the flake-like particles.

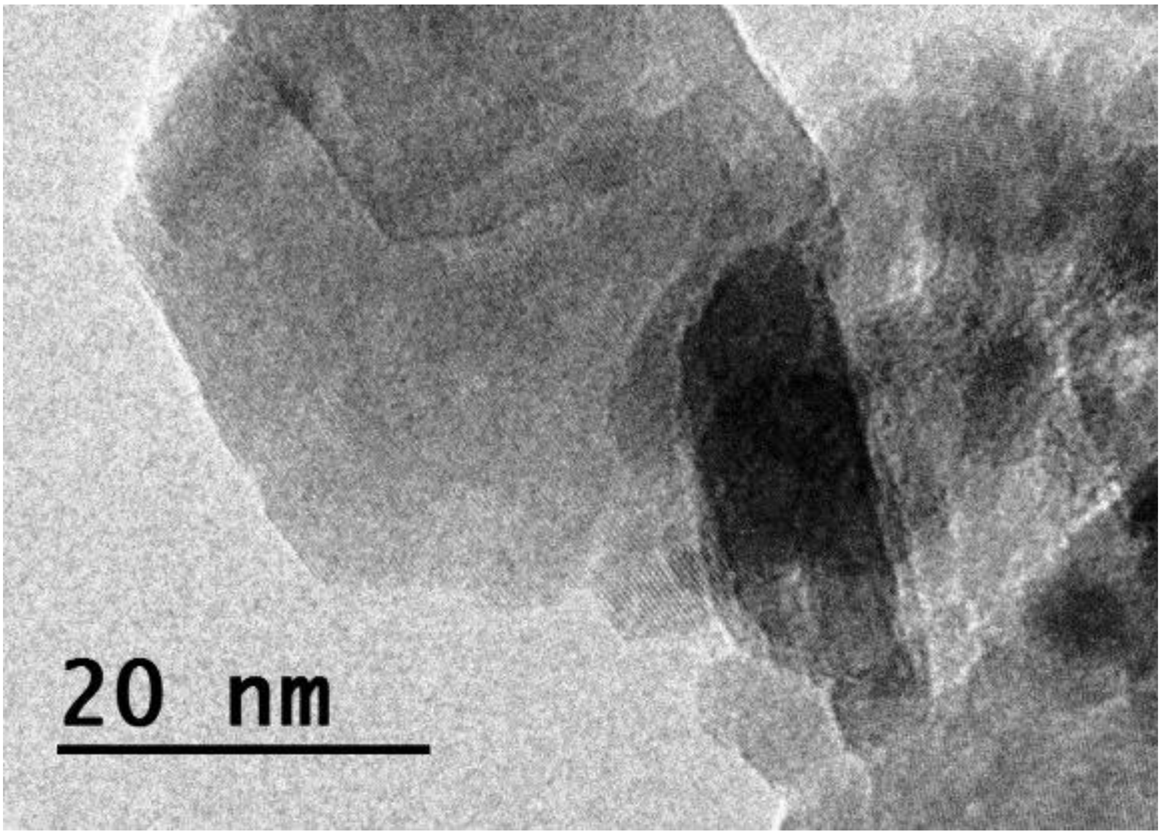

High resolution image showing the crystalline planes among the flake-like particles. This image is a zoom of Figure 3 of the main text.

Figure S2. TG-DTA (thermogravimetric-differential thermal) analysis and BET analysis of ZnO nanoparticles synthesized with 0.63 wt% PAAH of different molecular weights (2,000; 5,000; 100,000 and 240,000 g.mol^{-1}).

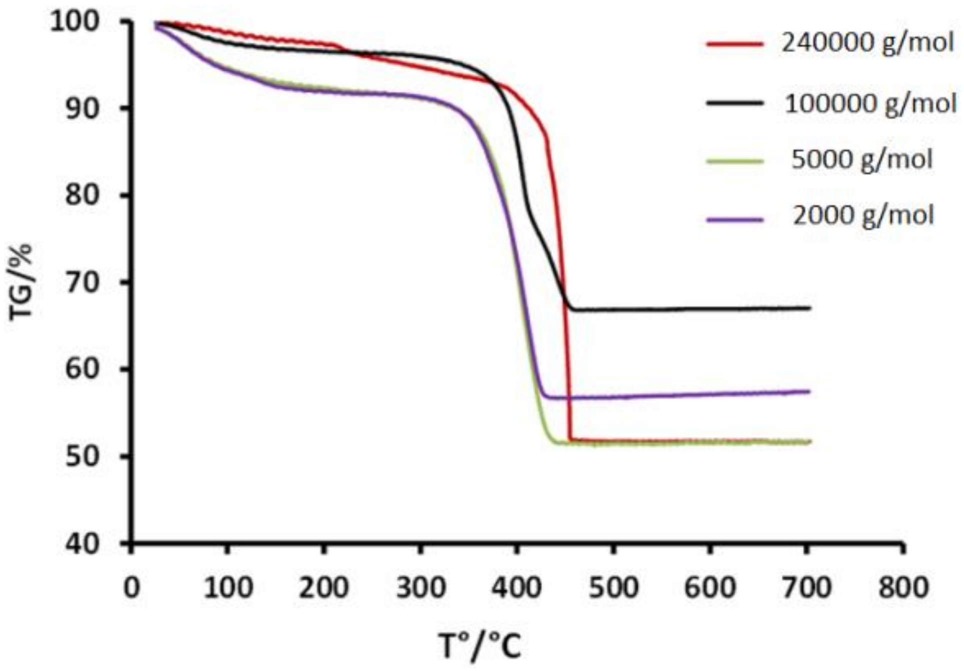

Table S1

Molecular weight (g.mol^{-1})	Relative weight loss in the range 20-150°C in %	Relative weight loss in the range 150-500°C in %	Specific area (m^2.g^{-1})	PL QY (%)
2,000	**7**	**36**	**21**	**19 ± 5**
5,000	**7**	**42**	**23**	**30 ± 1**
100,000	**2**	**32**	**51**	**15 ± 1**
240,000	**3**	**49**	**36**	**9 ± 1**

Both the TG-DTA and the BET analyses show no particular trend as a function of the PAAH molecular weight. The specific area is not affected significantly. A modification of the specific area cannot be evoked to explain the variation of the PL QY.

Figure S3: TEM images of the ZnO/PAAH composite synthesized by the hydrolysis of $ZnEt_2$ using a solution of 0.63 wt% of PAAH.

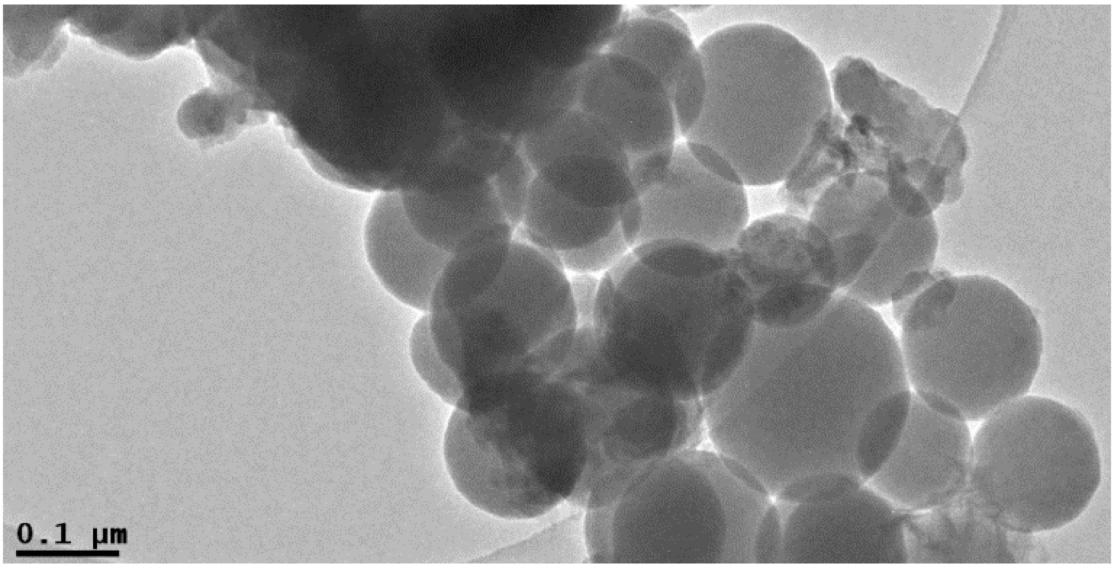

Low magnification image of the mesospheres made of the ZnO/PAAH composite.

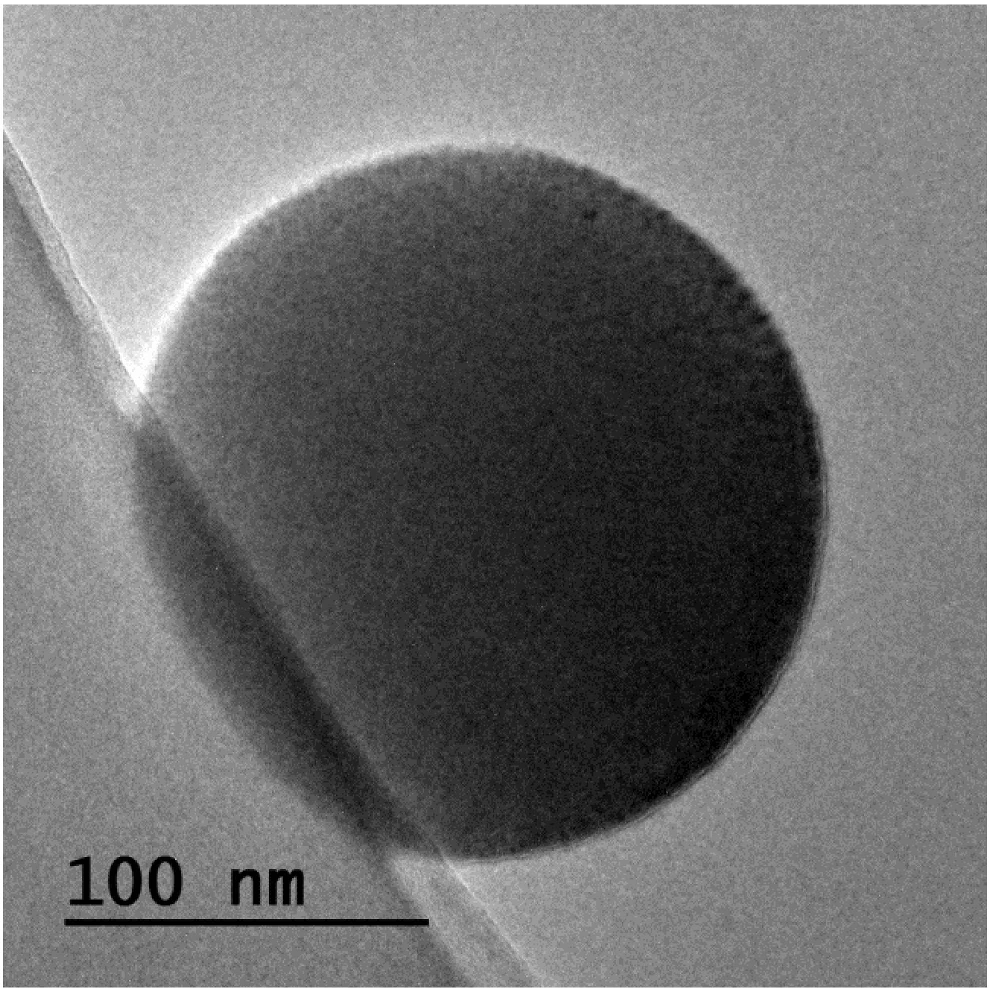

High magnification image of a mesosphere made of the ZnO/PAAH composite. Some ZnO nanoparticles are visible as dark spots.

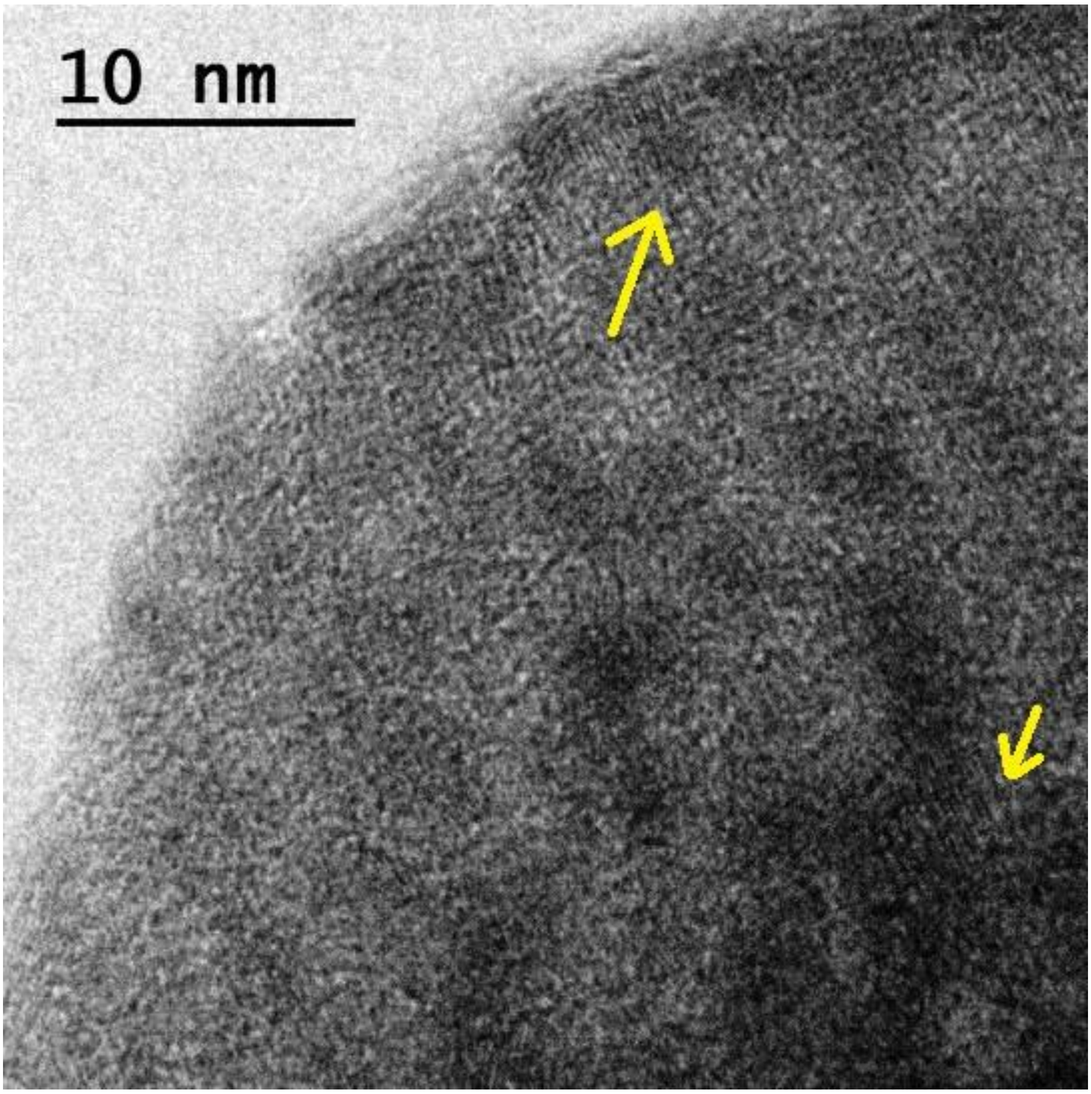

High resolution TEM image of a PAAH/ZnO mesosphere. The arrows indicate regions were the crystalline phase of ZnO nanoparticles can be identified, indicating that these nanoparticles are well crystallized.

Figure S4. PL spectrum of PAAH.

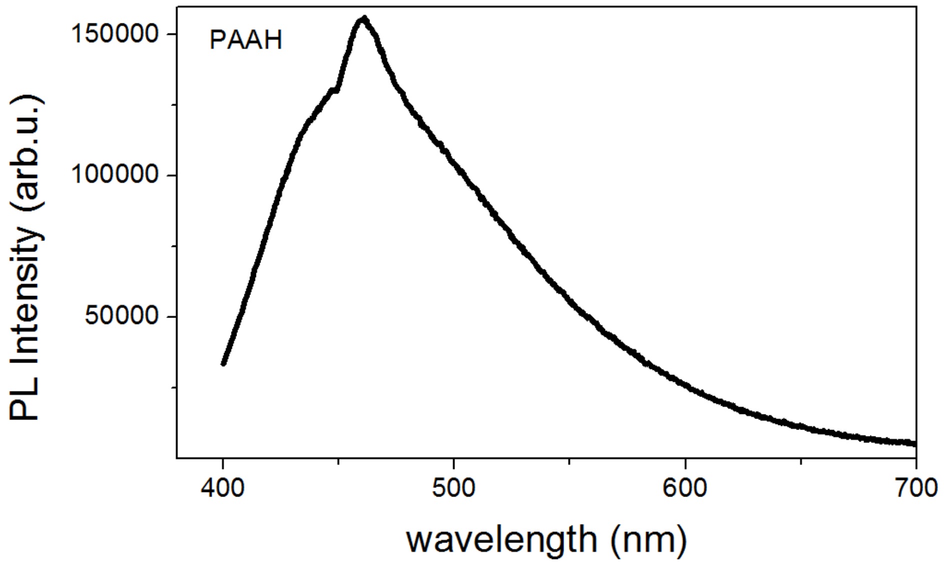

The PL spectrum of pure PAAH shows a peak at ~460 nm. As stated in the main text, this contribution is hardly distinguished, meaning that PAAH does not participate significantly to the PL of the samples.

Figure S5. Reflection spectrum (a) and bandgap estimation via the Kubelka-Monk approximation (b) of ZnO nanoparticles synthesized by the hydrolysis of the zinc diethyl with 0.63 wt% of PAAH.

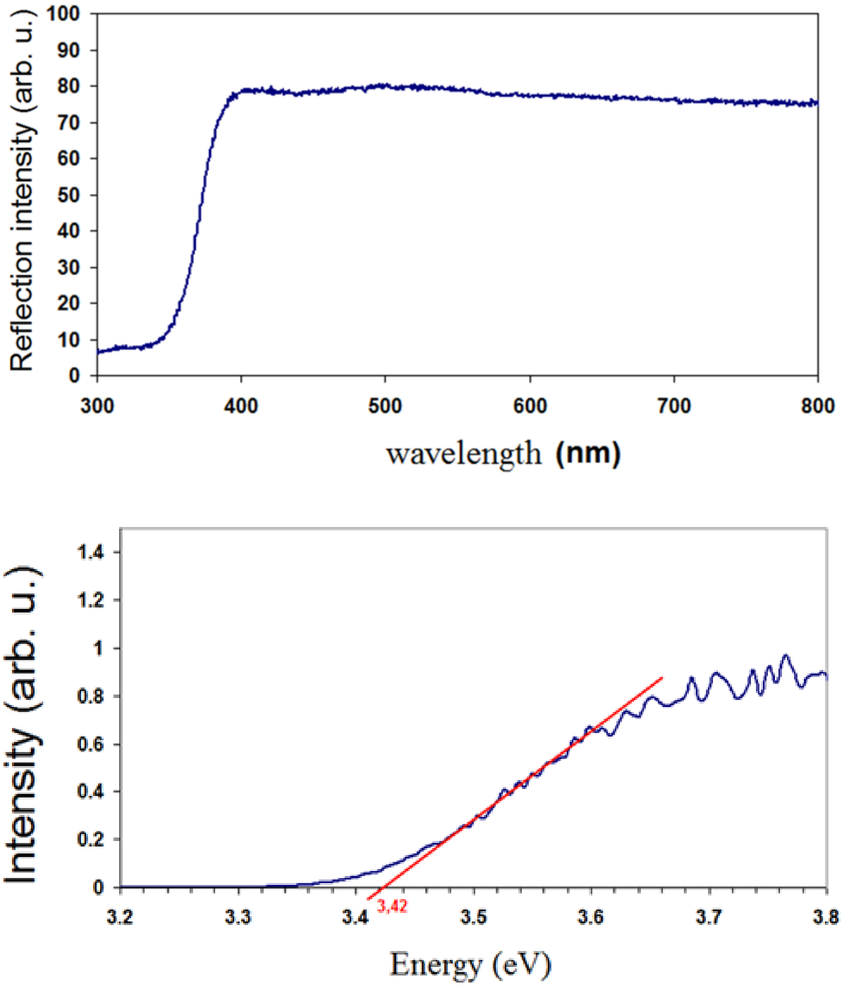

The band gap of ZnO NPs synthesized with 0.63 wt% of PAAH is 3.42 eV, as deduced using the Kubelka-Monk approximation from the reflection spectrum. This value is larger than that of bulk ZnO (3.37 eV at room temperature [1]) but in accordance with the PLE threshold shown in Figure 6. Actually, the real bandgap of ZnO is equal to that of bulk ZnO (no quantum confinement effect, but the Kubelka-Monk approximation applied to the reflection spectrum mainly highlights the major threshold due to the presence of PAAH).

Figure S6. PLE spectra of PAAH.

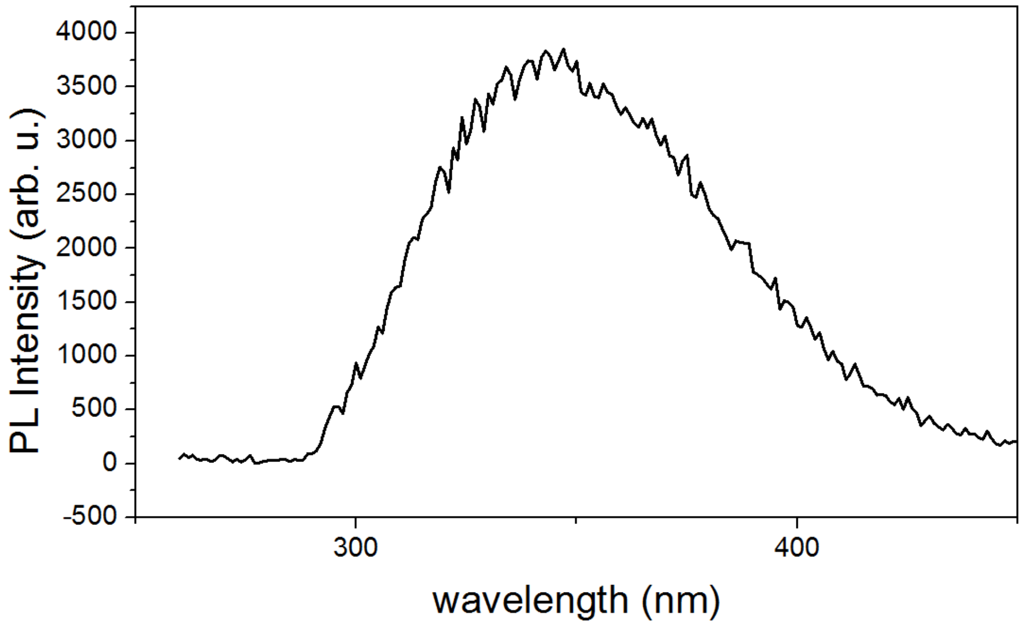

The PLE spectrum of PAAH shows a peak at 343 nm. Thus the gap energy between the highest occupied molecular orbital (HOMO) and the lowest unoccupied molecular orbital (LUMO) of PAAH can be deduced to be 3.6 eV.

Figure S7. FTIR spectrum (a), Raman spectrum (b), PL spectrum with PL QY (c) of ZnO nanoparticles synthesized by the hydrolysis of the zinc acetate with 0.63 wt% of PAANa and (d) Raman spectrum of ZnO nanoparticles synthesized by the hydrolysis of the zinc acetate with no surfactant.

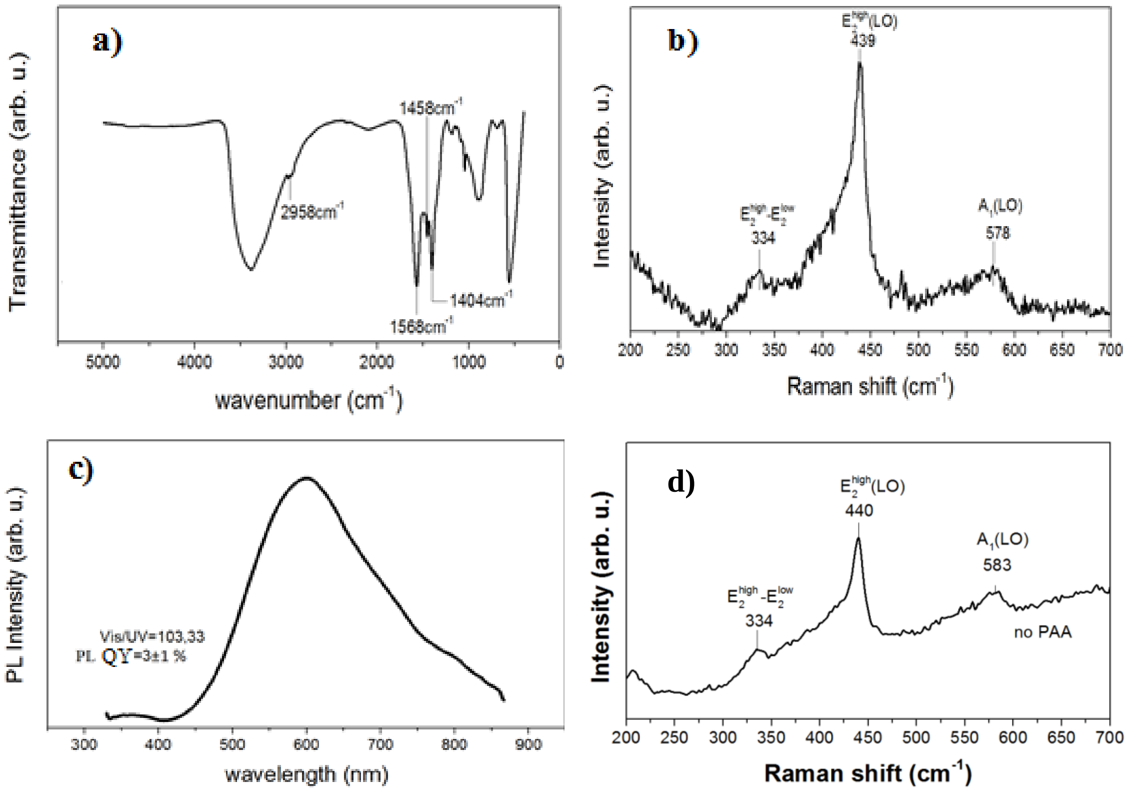

In the FTIR spectrum in Figure S7a), a broad band near 3400 cm^{-1} is observed, corresponding to the O-H stretching mode. Peaks located at 2958 cm^{-1} and 1458 cm^{-1} are attributed to the C-H stretching and bending modes of PAA^{-}, respectively, and the peaks located at 1568 cm^{-1} and 1404 cm^{-1} to characteristic asymmetric and symmetric stretching modes of the carboxylate ligands COO^{-}, respectively.[2] It confirms the adsorption of COO^{-} on ZnO NPs surface via covalent bond, but the quantity is much smaller relative to that of the OH group as compared to the case of ZnO NPs synthesized with PAAH. Comparing the Raman spectra of ZnO synthesized with PAANa and no organic acid in Figure S7b) and d), respectively, all ZnO NPs have similar modes. These modes are typical of hexagonal wurtzite structure of ZnO. They are located near 334 cm^{-1}, 440 cm^{-1}, 580 cm^{-1} and can be attributed to multiphonon modes, E^{2}_{high} mode and A_1(LO) mode, respectively [3]. This goes to show that all the ZnO NPs synthesized whether with PAANa or with no organic acid have a good crystalline quality and that the use of PAANa has no influence on the crystalline quality of ZnO NPs. The same conclusion holds for the optical properties (spectrum, Vis/UV and PL QY in Figure S7c)). Therefore, at this stage, the PAANa has no effect and it can be concluded that the direct adsorption of COO^{-} is not favorable for a high PL QY.

Figure S8. TEM analysis of the hybrid nanocomposite made of ZnO and short chain PAAH/PAANa (3:1) at a concentration of 0.63 wt%.

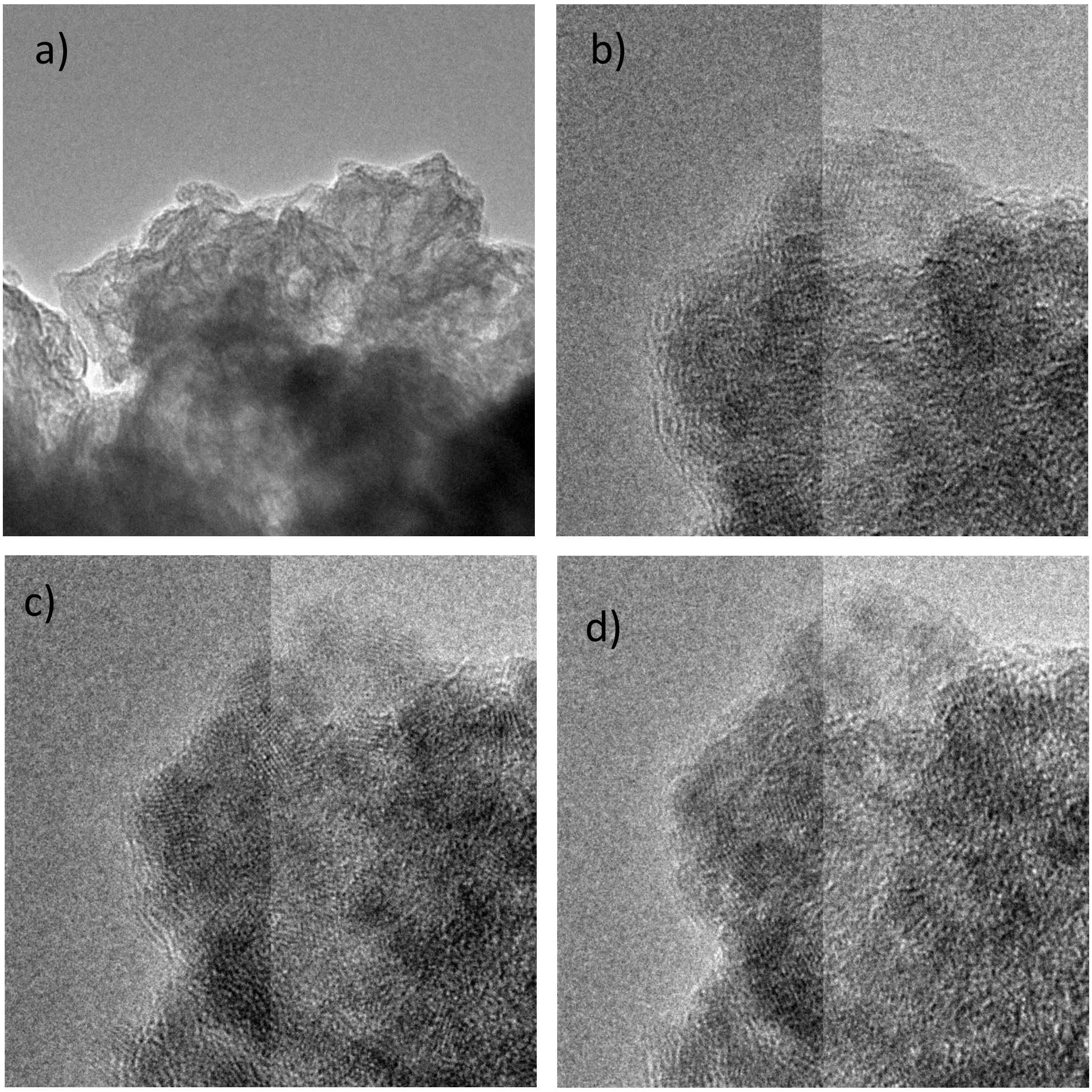

a) Low magnification TEM image of the ZnO-PAAH nanocomposite; b) HRTEM image of a zoomed region; c) and d)) HRTEM image of the same zoomed region several minutes after. Under electron illumination, the polymer phase undergoes irradiation damage. The polymer degradation leads to a better resolution of the diffracting planes among ZnO nanocrystals. The sample is thus made of both ZnO nanoparticles and the PAAH coating.

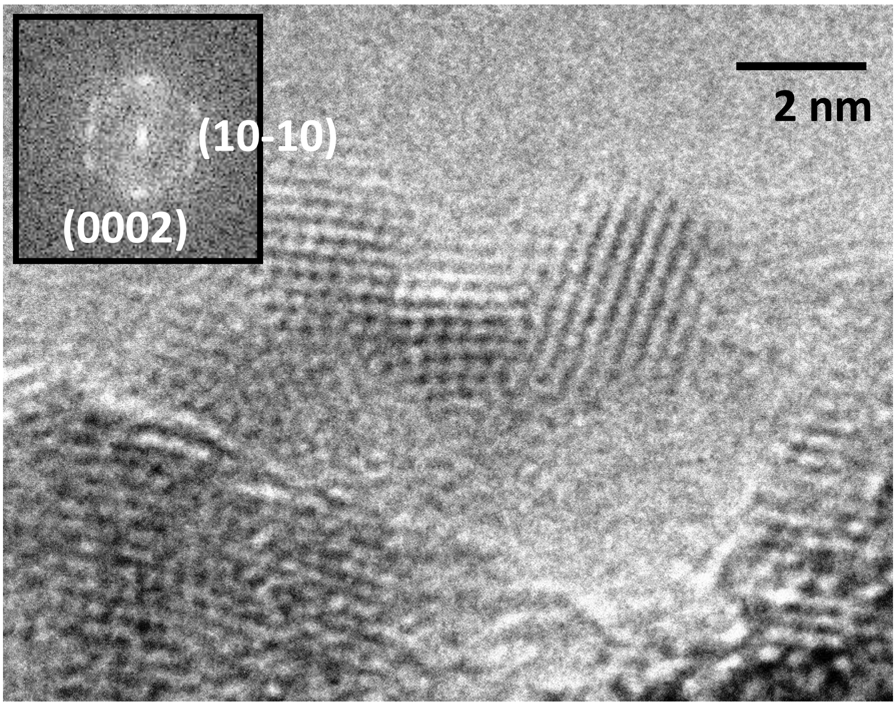

HRTEM image of some nanoparticles. The reticular planes of ZnO wurtzite are unambiguously distinguished (see inset, FFT of the image). The particles are quite small.

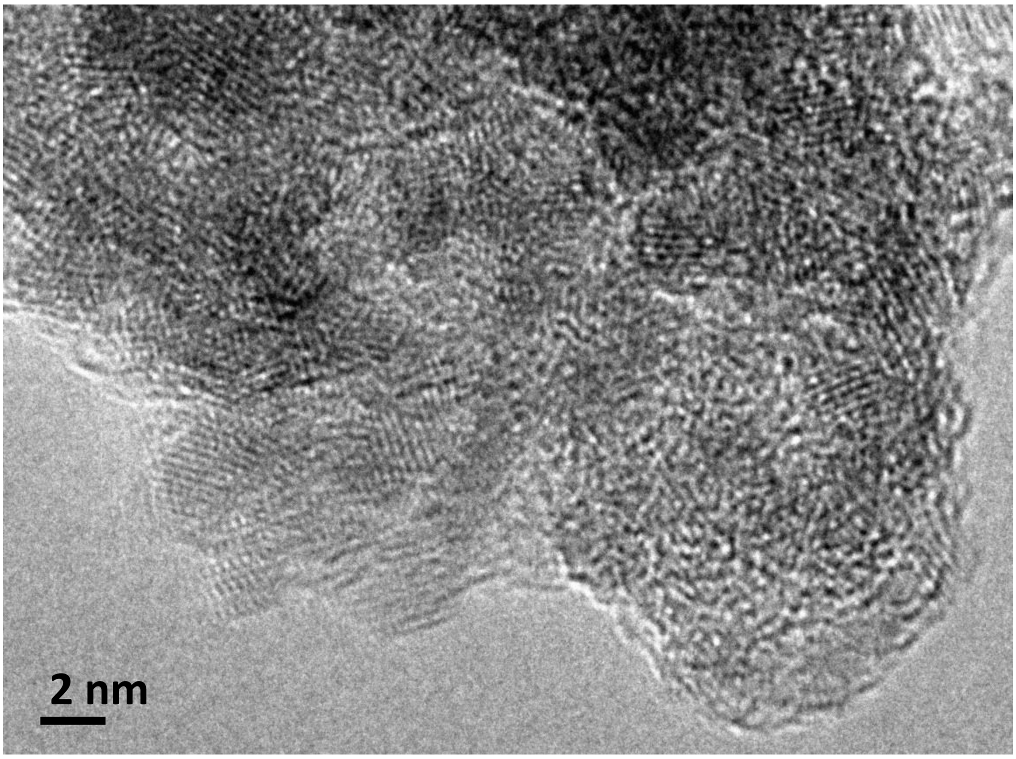

Numerous nanocrystals embedded in polymer. Some show two crystalline planes corresponding to the wurtzite structure of ZnO. Others just show one type of crystalline plane with a reticular distance between the (1000) and (0002) ones, consistantly with what is seen in the diffractograms.

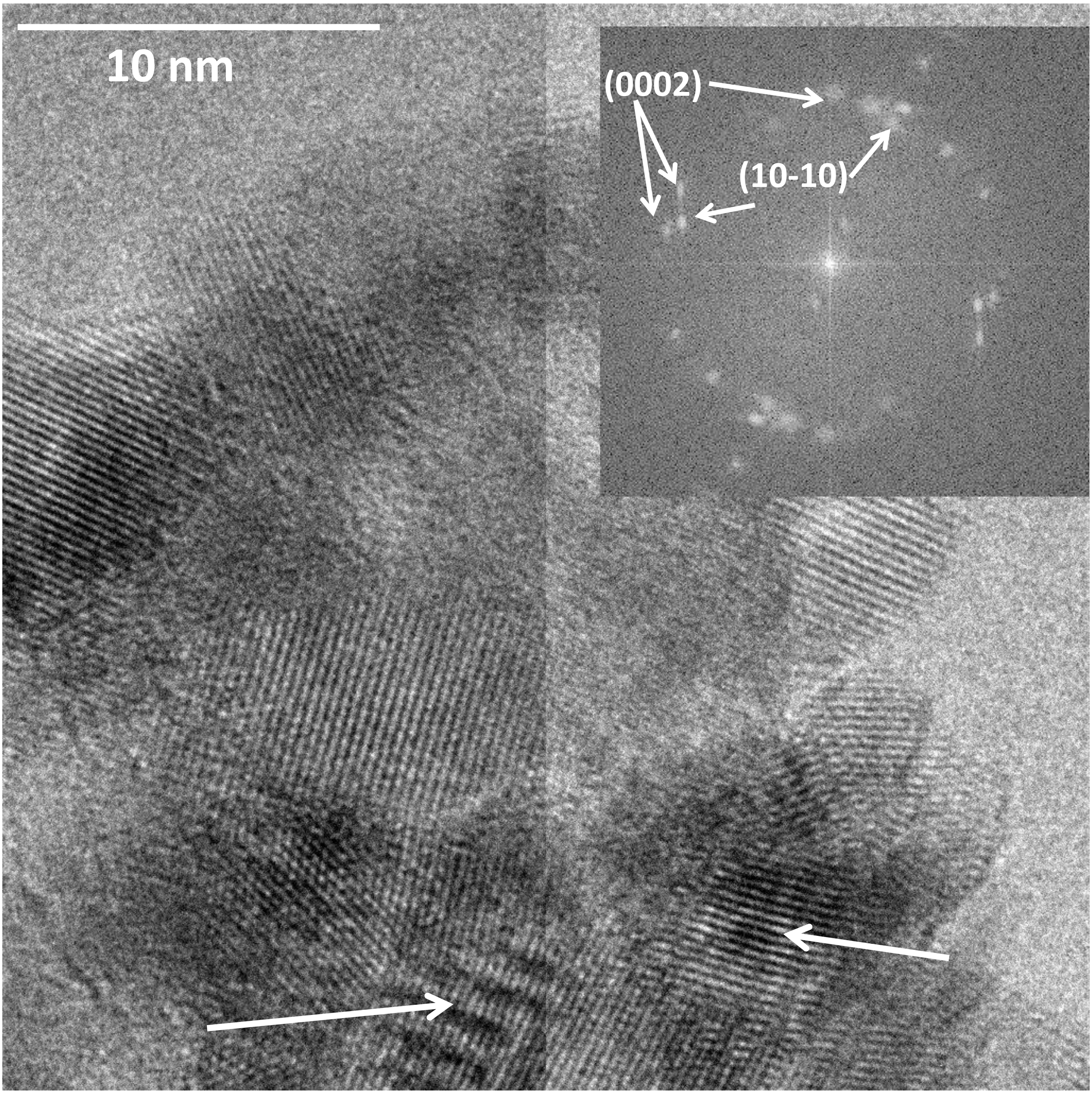

The HRTEM image shows many nanocrystals with one diffracting family plane. The diffracting pattern assigned to the (0002) planes of the wurtzite structure could be assigned to the $Zn(OH)_2$ wulfingite as well. Nevertheless, some nanocrystals appear distorted (see arrows).

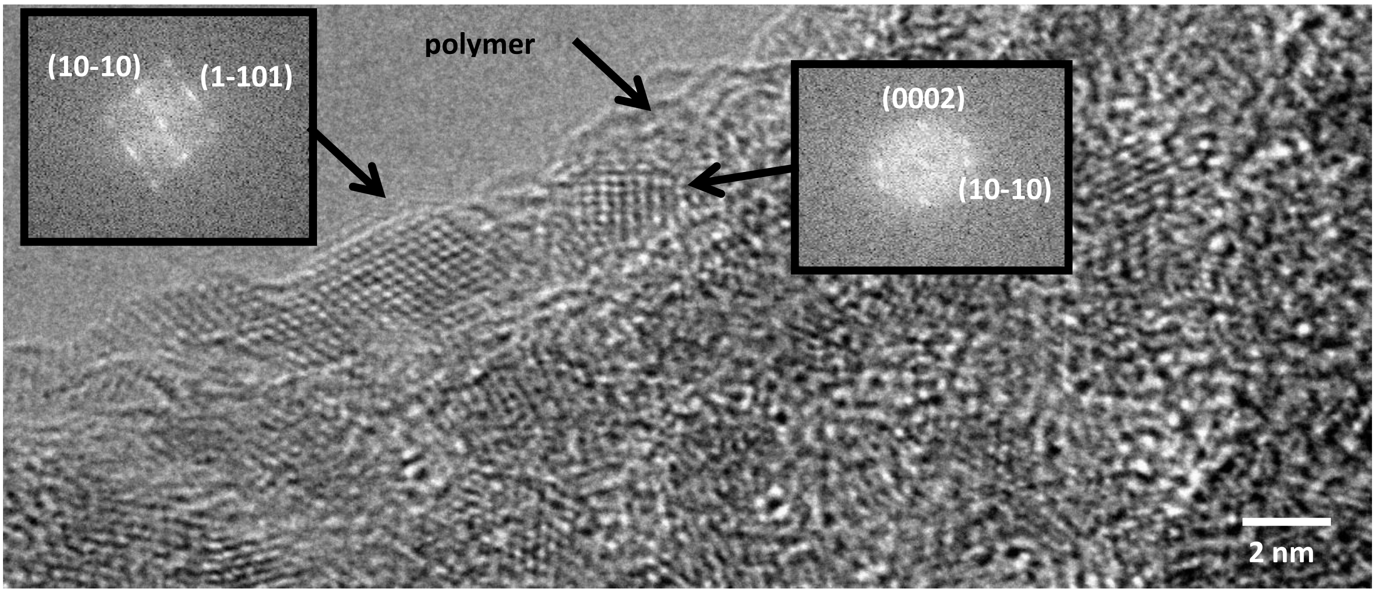

HRTEM of small ZnO particles. The wurtzite structure can be observed but is distorted: the (1-101) distance is 2.6 Å instead of 2.48 Å; the angle between the (1-101) and (10-10) planes is about 118° instead of 116.1°.

1. Ü. Özgür, Y. I. Alivov, C. Liu, A. Teke, M. A. Reshchikov, S. Doğan, V. Avrutin, S. -J. Cho, H. Morkoç, *J. Appl. Phys.*, **2005**, *98*, 041301.

2. F. Jones, J. B. Farrow, W. van Bronswijl, *Langmuir*, **1988**, *14*, 6512.

3. T. C. Damen, S. P. S. Porto, B. Tell, *Phys. Rev.*, **1966**, *142*, 570.
